# Robust Data Integration Method for Classification of Biomedical Data

**DOI:** 10.1007/s10916-021-01718-7

**Published:** 2021-02-23

**Authors:** Aneta Polewko-Klim, Krzysztof Mnich, Witold R. Rudnicki

**Affiliations:** 1 Institute of Computer Science, University of Bialystok, Bialystok, Poland; 2 Computational Center, University of Bialystok, Bialystok, Poland

**Keywords:** Random forest, Data integration, Feature selection, Biomedical data

## Abstract

We present a protocol for integrating two types of biological data – clinical and molecular – for more effective classification of patients with cancer. The proposed approach is a hybrid between early and late data integration strategy. In this hybrid protocol, the set of informative clinical features is extended by the classification results based on molecular data sets. The results are then treated as new synthetic variables. The hybrid protocol was applied to METABRIC breast cancer samples and TCGA urothelial bladder carcinoma samples. Various data types were used for clinical endpoint prediction: clinical data, gene expression, somatic copy number aberrations, RNA-Seq, methylation, and reverse phase protein array. The performance of the hybrid data integration was evaluated with a repeated cross validation procedure and compared with other methods of data integration: early integration and late integration via super learning. The hybrid method gave similar results to those obtained by the best of the tested variants of super learning. What is more, the hybrid method allowed for further sensitivity analysis and recursive feature elimination, which led to compact predictive models for cancer clinical endpoints. For breast cancer, the final model consists of eight clinical variables and two synthetic features obtained from molecular data. For urothelial bladder carcinoma, only two clinical features and one synthetic variable were necessary to build the best predictive model. We have shown that the inclusion of the synthetic variables based on the RNA expression levels and copy number alterations can lead to improved quality of prognostic tests. Thus, it should be considered for inclusion in wider medical practice.

## Introduction

The cancer pathophysiology is related to both genetic and epigenetic changes that are described by various types of biological data. Each type of cancer is very complex, with high variability of sources, driver mutations, and responses of the host to therapy [1]. Therefore, commonly used clinical data does not provide all the information necessary for analyses and predictions [2, 3].

In recent years, high-throughput omics data delivered novel biological insights into human biology and health [4]. The omics data was successfully utilized for different goals, such as cancer outcome prediction, survival prediction, prediction of response to a pharmaceutical compound, risk stratification, and clustering of cases [5–7]. However, integration of various types of omics data [4, 8], and the integration of omics and non-omics data [9–11] is necessary to gain a deep understanging of biological systems. For this purpose, many different modeling approaches have been proposed [8, 12]. Machine learning methods in particular, are crucial to the integrative analyses due to the constantly growing dimensionality of omics data [13, 14].

This study is focused on the prediction of the clinical endpoint with clinical and molecular genomic data. The prediction of clinical endpoints and clinical outcomes based on the molecular data for cancer patients is extremely difficult [3, 15]. Nevertheless, predictive and diagnostic models significantly increased the efficiency of diagnostics, prognostics, and therapeutics in patients with cancer [15, 16].

With the advent of new types of molecular data, more effective prediction of clinical endpoints requires new methods for integration of clinical, as well as multiple types of molecular data [17]. The comprehensive pan-cancer analysis of multiple omics profiles and clinical factors, conducted by Zhu et al. [17] showed that data integration improved prognostic performance in 7 out of 14 of cancer types examined, when compared with the use of clinical variables alone.

The integration of clinical and omics data is challenging due to the heterogeneity of the data sets [3, 18]. Clinical data consist of a few variables, strongly related to the analyzed phenomenon. Conversely, in omics data sets the signal is distributed through many weak variables. Three major strategies are generally used to deal with heterogeneous data [3, 19, 20].

In the early clinico-genomic integration strategy (also referred to as concatenation-based integration), one combines several data sets and relies on the machine learning algorithm for finding meaningful relationships across data sets boundaries. In this approach the model building is usually preceded by a feature selection (FS) step [3], due to a very large number of variables. The feature selection procedure is performed either for molecular data only [21], or on the combined dataset [22]. In the late integration strategy (model-based integration) is conducted in two stages. First, separate models are built for each data set. Then, their results are used as input for the second level machine learning algorithm. These two strategies can be generally executed using standard machine learning algorithms.

In the third approach, the intermediate integration strategy (transformation-based integration), each individual data set is transformed into an intermediate representation (e.g. network, kernel) that preserves the individual properties of the data set. The intermediate representations of data sets are merged before developing prediction models. The advantages and disadvantages of these approaches are thoroughly discussed in [3].

One can observe that none of the above strategies is appropriate for strongly heterogeneous data [3, 23, 24]. A handful of strong clinical variables is incomparable to many weak omics ones, which makes the early and intermediate integration inefficient. The late integration is also problematic due to the different performance of classification based on such different data. In some cases a single important clinical feature delivers similar information on the analysed phenomenon as the entire whole molecular data set. Hence, the natural way to integrate such data, is to build an aggregate of omics data and treat it as an additional clinical feature.

In the current study, a novel methodology for integrating various types of molecular data with clinical data is proposed. It is a mixture of early and late integration strategy and can be performed using standard machine learning algorithms. First, independent predictive models are developed for each type of molecular data. The results of these models can be treated as synthetic features that are complex aggregates of many molecular variables, maximising the information on the investigated phenomenon. Then, these new synthetic variables are included in the set of clinical descriptors. Finally, a machine learning model is built using the extended data set, consisting of the clinical and synthetic molecular variables.

The methodology outlined above was applied to predict clinical endpoints for breast cancer patients (BRCA data set) or urothelial bladder carcinoma cancer patients (BLCA data set). Both types of cancer were already investigated in many studies in the context of data integration for the prediction of the clinical endpoint [18, 25, 26]. The current work is an extended and improved follow up of the earlier pilot study performed for the BRCA data set, using two FS methods [24].

The main contributions of the current study are as follows:

- a novel approach to the problem of integration of diverse biomedical data of cancer patients;
- comparison of the performance of single predictive models with combined models;
- demonstration that synthetic molecular variables (i.e. classifier outputs) may be robust prognostic markers.

## Materials and Methods

All data processing and analysis were conducted by using R version 3.4.3 [27] and R/*Bioconductor* packages [28].

### Data sets

#### Breast cancer

Three types of descriptors were available for breast cancer patients: clinical data (*CD*), gene expression profiles (*GE*) obtained with Illumina Human HT-12 v3 microarray, and copy-number alterations data (*CNA*) obtained with Affymetrix SNP 6.0. The data was obtained from the Molecular Taxonomy of Breast Cancer International Consortium (METABRIC) project [29].

##### Clinical data

The clinical data set includes twenty five clinical features, obtained from the diagnostic tests, such as: *Prosigna, Breast Cancer Index, EndoPredict, MammaPrint, Mammostrat*, and *Oncotype DX DCIS*. All samples with missing values were removed, with the exception of the *tumor stage* feature, where the null value was replaced by 0. All qualitative clinical data was converted into numerical data. The disease-specific survival was used as the clinical endpoint (decision variable), since it predicts breast cancer survival more accurately than the overall survival [30].

##### Molecular data

The primary gene expression set contains 1906 samples described by 24369 continuous variables corresponding to the gene expression levels. The CNA set contains 1483 samples described by 22544 discrete variables, corresponding to alterations of the number of copies of genes. The missing values of probes were replaced by mean and median values, for GE and CNA sets respectively. Additionally, the filtering of variables was performed for the GE set, based on the quality of the signal. Two criteria were used - a sufficiently high intensity and variation of the signal. The low intensity of measured gene expression is generally considered as noise. What is more, gene expression should have a sufficiently high variation to be included in analysis - the small changes of activity, even if statistically significant, are very unlikely to have any biological relevance. Filtering was performed with the help of a dedicated function from *genefilter* of *Bioconductor* package [31]. The intensity threshold was set at the first quartile of the distribution of the maximum gene expression levels. Only genes for which at least 10% of samples have intensity greater than this threshold were included. As for the variation criterion, only the genes for which the robust coefficient of variation [32] was higher than 0.05 were included. This pre-filtering procedure limited this number of GE features to 8673.

The complete records, comprising of CD, GE and CNA data, were available for 1394 patients (781 survivors and 613 deceased), and were used in the study as the BRCA data set.

#### Urothelial bladder carcinoma cancer

Four types of data were available for these patients: clinical information, the median mRNA levels of gene expression (RNA-seq V2 RSEM normalized expression values), DNA methylation profiles (METH) generated from Illumina HM450K array (beta-values for genes), and protein expression profiling with reverse-phase protein arrays (RPPA). Data was obtained from the Cancer Genome Atlas Urothelial Bladder Carcinoma (TCGA-BLCA) program [33].

##### Clinical data

The primary clinical data set contains twenty one clinical features, comprising of individual patients’ information, such as demographic characteristics, cancer topography and morphology, and treatment information. The samples with incomplete information were removed from the original set. Both the nominal, and the ordinal data were transformed into numerical representation.

##### Molecular data

The primary RNA set consists of 408 samples and 20437 probes, the METH set of 413 samples and 16221 probes, and the RPPA set of 344 samples and 225 probes.

The data preparation procedure was performed for all molecular data. First, the missing values of variables were replaced by a their mean value. Then, all values were replaced by their logarithm in base 2. Next, the initial prefiltration of low-intensity probes and probes with low variability across samples was performed with the help of the *genefilter* package, in the same way as for BRCA data set. The ComBat function with *sva* R package [34] was used for removing batch effects between samples with different tissue source sites.

After integrating clinical and molecular data sets, the final BLCA data contained records of 320 patients (149 survivors and 171 deceased) in four subsets containing 21 clinical descriptors (CD), 19006 mRNA gene expression profiles (RNA), 15628 DNA methylation profiles (METH), and 219 reverse-phase protein profiles (RPPA).

### The cross-validation procedure

The goal of the current study requires an unbiased estimation of the quality of predictions for all the machine learning models developed. Therefore, the entire machine learning pipeline, including feature selection and model building for molecular data sets, the final data integration and testing the quality of predictions, was conducted within a repeated *k*-fold cross validation procedure. The general cross-validation protocol is shown in Algorithm 1. In this study, we conducted *r* = 30 repeats of *k* = 5-fold cross validation.

### Feature selection methods

#### Clinical data

Clinical descriptors are highly diverse, some of them correspond to numerical values resulting from measurements, some to ordinal values and some to categorical descriptions. Therefore, the CD set is not suitable for analysis with tests that require numerical data. Instead, the all-relevant FS algorithm Boruta [35, 36], was used to identify and the relevant clinical variables. This selection was performed only once, using the entire data set. The resignation from cross-validation, could lead to *positive* bias in the estimated quality of models based on CD data only. Consequently, the estimate of improvement due to expanding the description by adding molecular data to clinical variables, may have a small *negative* bias. Nevertheless, the bias is expected to be small, due to the small number of clinical variables and a sharp border between relevant and irrelevant ones. Thus, we use this approach, as it is both simpler for interpretation and computationally less expensive. 
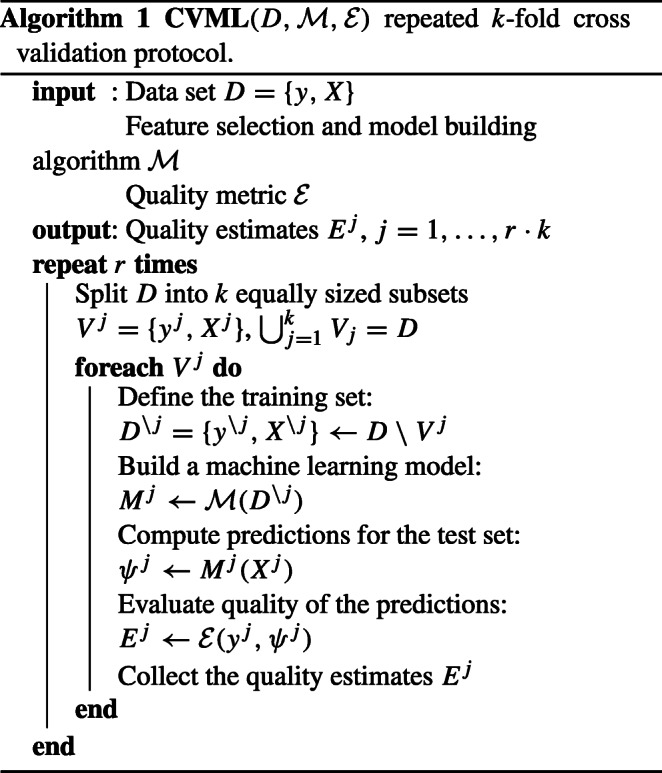


#### Molecular data

It is known that the filtering FS methods have better generalisation properties than wrappers and embedded methods [37], because they are not related to the algorithms used later for classification. Therefore, two FS filters, namely Mann-Whitney U-test [38] and MDFS [39] were applied for detecting the informative features in molecular profiling data sets. The former is a standard R library, for the latter we used *MDFS* R package version 1.0.5 [40]. In both cases, feature ranking was based on the p-values of the tests. P-values were corrected for multiple testing with the help of the SGoF procedure [41].

##### U-test

The U-test is the nonparametric equivalent of the two-sample t-test that assigns a probability to the hypothesis that two independent samples corresponding to two decision classes (vital status of patients: death/alive) are drawn from populations with the same average value. Application of the U-test is recommended when the data does not conform to a normal distribution and/or the sample sizes are small. Both these cases commonly occur for molecular data.

##### MDFS

The MDFS algorithm measures the decrease of the information entropy of the decision variable due to knowledge of *D*-dimensional tuples of variables and measures the influence of each variable in the tuple In this study, two versions of MDFS algorithm (1D and 2D) were used, referred to as MDFS-1D and MDFS-2D, respectively. 
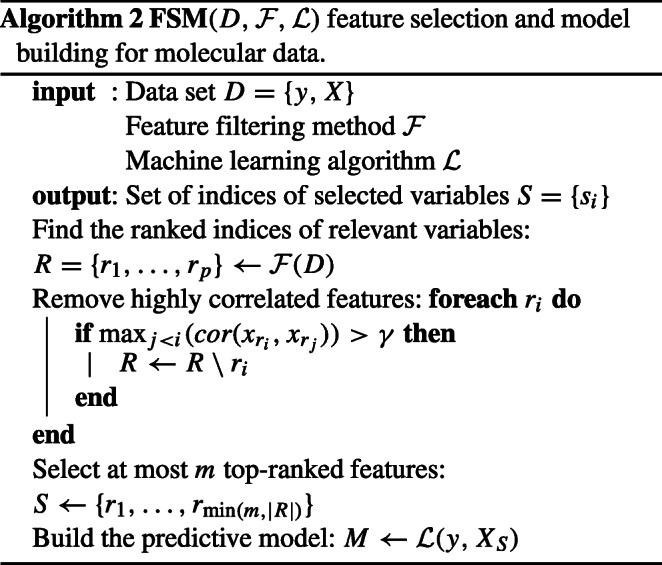


##### Redundancy removal

Molecular profiling data contains multiple highly correlated features, that can have an adverse effect on classification accuracy and therefore the greedy algorithm was used to remove redundant features. The final set of features was determined by removing features that were highly correlated with higher-ranking ones. The cut-off level of Spearman’s rank correlation coefficient was set to 0.7. The number of features from all molecular sets used for model building, was limited to *m* = 100. This value was established experimentally by comparing the quality of the models as a function of *m*. The algorithm 2 describes the entire feature selection and model building procedure for molecular data.

### Machine learning methods

#### Classification algorithm

The predictive models were built using the random forest algorithm [42], implemented in *randomForest* R package [43]. Random forest is an ensemble of decision trees, where each tree is built on a different bagging sample of the original data set. For each split, a subset of variables is selected randomly and the one is selected that allows to achieve the highest Gini coefficient for the resulting leaves. Random Forest works well on data sets with a small number of objects, has few tunable parameters that don’t relate directly to the data, and very rarely fails. It usually gives results that are either best or very close to the best results achievable by any classification algorithm, as shown in comparison of performance of 179 algorithms on 121 data sets performed by Fernandez-Delgado et al. [44]. Being a tree-based algorithm, random forest is insensitive to the type of data – it can deal with binary, categorical, as well as continuous variables. Hence, it is particularly useful in the analysis of clinical data, that contain all the types of variables. Another advantage of the random forest algorithm is the built-in OOB mechanism for unbiased estimation of predictions (the OOB is an acronym for out of bag). It is obtained by using the objects, which were not included in the bagging sample used for the tree building, to test the quality of the predictions. Each object is “in the bag” for 1 − *e*^− 1^ fraction of trees and is OOB for *e*^− 1^. All trees for which the object is OOB make prediction of its class. These predictions are then counted and the predicted class is assigned using the same criteria that would be used for predicting class for new data. However, in this case we already know the class of all objects, hence we can compute all quality measures, such as the error rate, the AUC or the MCC. This measures are in most cases equivalent to the external cross-validation. Using OOB estimates allows to simplify the data integration procedure, see remarks in the *Proposed integration strategy* subsection.

#### Evaluation metrics

The quality of the models was evaluated using three metrics: the accuracy (ACC), the area under the receiver operator curve (AUC), and the Matthews correlation coefficient (MCC) [45]. It should be noted that the MCC and AUC metrics are better suited to evaluate the quality of a classifier for the unbalanced population than the simple ACC. Hence only the MCC and AUC are used for comparisons, whereas the ACC is only reported for completeness of results. For random forest classification, the values of MCC and ACC metrics depend on the value of *cutoff* hyperparameter, i.e. the number of votes that lead to a choice of a decision class. In this study, the hyperparameter was tuned to maximize MCC, using OOB estimate of predictions for the training data.

### Data integration

In this study, three strategies for the integration of clinical data with molecular data sets for clinical endpoint prediction were explored.

#### Early integration strategy

In the initial approach, all the relevant clinical features with top-*m* most relevant features obtained from a given molecular data set were simply merged. Unfortunately, such an approach did not improve the results of the classifier based on clinical data alone. Weak molecular variables seemed to be ignored by the random forest algorithm in the presence of much stronger clinical ones. Therefore, alternative approaches were tested.

#### Late integration strategy

**Fig. 1:**
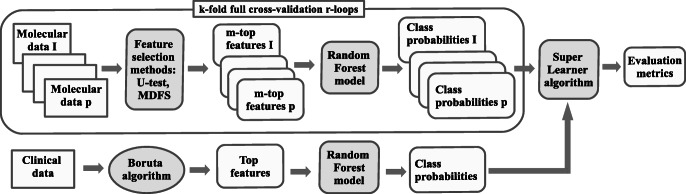
Procedure scheme for clinical endpoint prediction with clinical and molecular data using the super learner algorithm. See notation in text

The implementation of the late integration strategy is based on the super learning algorithm, proposed by Van der Laan et al. [46]. A general scheme of this protocol is displayed in Fig. 1.

The algorithm uses internal cross validation to obtain unbiased predictions of machine learning models for particular data sets. The vectors of cross-validated predictions are treated as new explanatory variables and used to build the second-order machine learning model. The super learning approach is universal: it allows to integrate diverse feature selection and machine learning techniques, as well as multiple data sets. We used it to combine all the individual sets of predictions for various data sets obtained with protocols using various feature selection methods. Hence, our second-order models were built using 7 synthetic variables for BRCA data, and 10 for BLCA data.

We applied three diverse methods to build the combined model: non-negative linear combination, random forest algorithm, and a simple mean of *k* best-performing base models. The last method, which may be identified with the “wisdom of crowds” principle [47], often performs as good as more complicated methods. To reduce the noise due to the random cross validation splits, we ran 30 loops of 5-fold internal cross validation to produce 30 separate super learning models. The eventual prediction was an average over all the combined models.

The well-established method for evaluating the quality of the final model involves an external cross validation. However, it is very demanding computationally. Instead, we applied Bootstrap Bias Correction algorithm that gives similar results with much smaller computational effort [48, 49]. The algorithm allows for unbiased quality estimation of the combined model, using only one run of the internal cross validation to compute base models. In this study, 30 repeats of bootstrap sampling procedure was used to estimate the performance of combined models.

#### Hybrid integration strategy

**Fig. 2:**
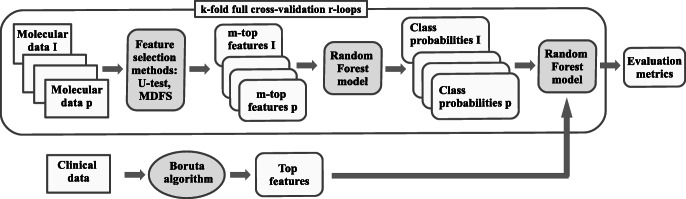
Procedure scheme for clinical endpoint prediction with clinical and molecular data using the proposed method. See notation in text

The hybrid data integration approach is based on combining clinical descriptors with synthetic features built as machine learning predictions for molecular data sets. The data integration procedure is presented in the Fig. 2 and in algorithm 3. The algorithm 4 describes the way of computing the combined predictions for new data.

The OOB estimates (see the description of random forest algorithm in the subsection *Machine learning methods*) of predicted probability of “positive” decision, obtained for molecular data, are used as synthetic variables. Hence, the new variables are represented by real numbers in from the (0,1) interval. Note that the present version of the algorithm bases on unbiased OOB prediction estimation, which is a built-in feature of random forest classifier. This allows for building the synthetic variables using the training data set. One can expect only a minor bias of the OOB predictions for molecular data due to the feature selection. This small bias may slightly overestimate the strength of the artificial molecular features. If another machine learning algorithm was used, the predictions on the training set could be strongly overfitted and thus incomparable to clinical variables. In such a case, cross-validated predictions should be used, like in the super learning procedure. 
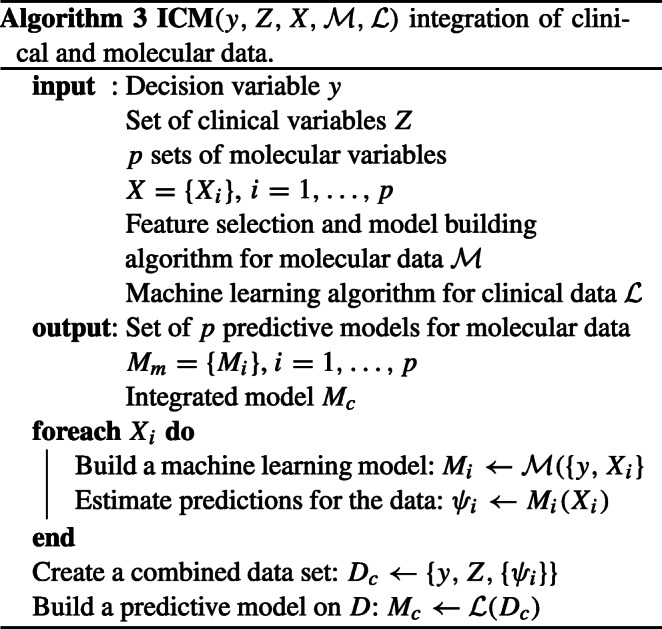

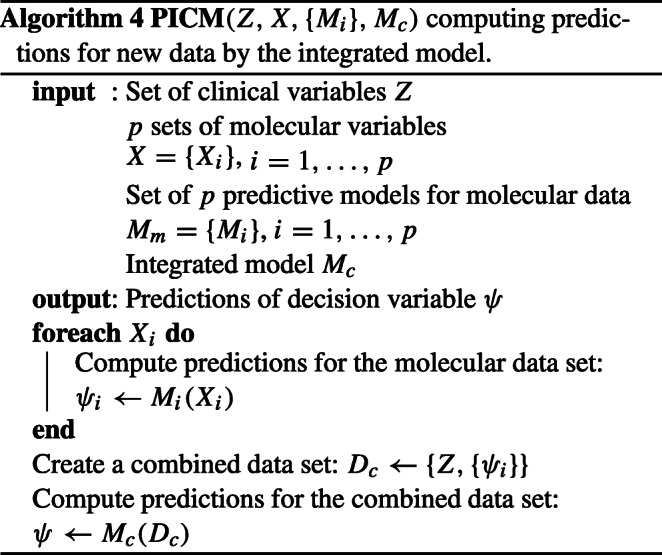


### Sensitivity analysis

A crucial advantage of the hybrid approach is the possibility of evaluating the contribution of particular molecular data sets to the information of the decision variable. This was achieved by performing sensitivity analysis of the predictive model to the removal of the descriptive variables. The predictive models were constructed using a data set with a single feature (clinical or synthetic) removed. In this way, the influence of the single feature on the quality of the model using all other informative features could be established.

The next step of the conducted analysis is similar to the well-known recursive feature elimination method. A series of predictive models was built. It started with models including all features. Then at each step the least important feature was removed and new model was built. Consequently we were able to build a well-performing model using a smaller number of features.

## Results

Four stages of the study generated different types of results. In the first stage we obtained information which clinical descriptors and which individual molecular variables carry information relevant for prediction of the clinical end point for BRCA and BLCA cancer. We also obtained the predictive models based on the individual data sets and compared their performance. In the second stage the individual models were combined using the late information strategy and we compared the results of the individual models with that of the combined model. In the third stage the hybrid integration approach was used for generation of the combined model, and contribution of different data sets to the final prediction was examined. Finally, in the fourth stage the influence of individual variables was examined and compact models were obtained based on the hybrid integration. The results of these stages are presented below. The effectiveness and performance of the compared protocols for integrating clinical data and high-dimensional molecular data were evaluated using the BRCA cancer data from the METABRIC project and BLCA cancer data from the TCGA project.

### Informative variables

#### Clinical variables

Boruta feature selection algorithm [50] was used to select informative clinical variables with BRCA and BLCA data. For BRCA data, the 17 of 25 clinical descriptors were deemed relevant: *intclust, cohort, age at diagnosis, NPI, ER IHC, breast surgery, three gene, claudin subtype, chemotherapy, radio therapy, grade, tumor size, tumor stage, ER status, HER2 status, PR status, oncotree code* (in order of their importance). Generally, the variables are weakly correlated with each other, except for one pair *ER IHC* and *ER status*, for which the correlation coefficient is *r* = 0.82.

**Fig. 3:**
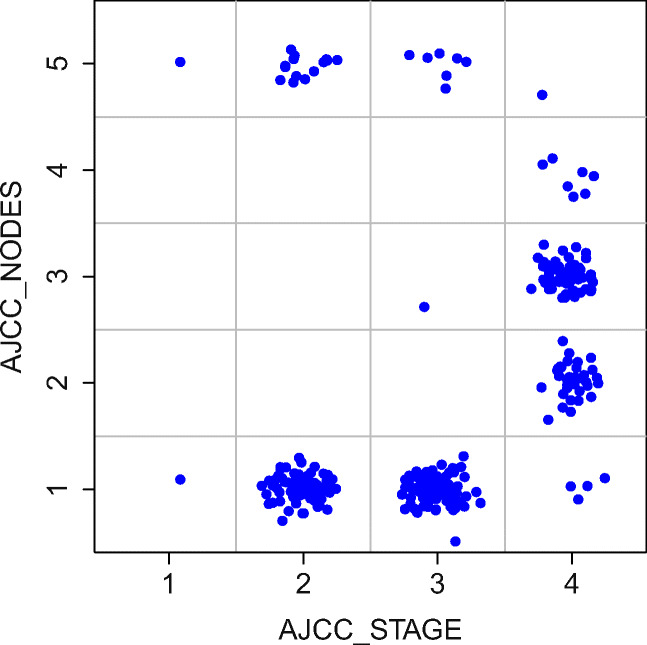
Statistics of values for AJCC_STAGE and AJCC_NODES variables from the BLCA clinical data. Despite the weak correlation, the variables are clearly interdependent. Use of both variables instead of one of them in the predictive model curtails its quality

In the case of BLCA data, only 5 of 21 clinical descriptors, namely *histological subtype (hist_subtype), age, ajcc_nodes, ajcc_stage,* and *grade* proved relevant. Correlations between the selected variables are weak, however, the variables *ajcc_nodes, ajcc_stage* are strongly related (see Fig. 3).

#### Molecular variables

Three feature filtering methods were used to identify the relevant molecular variables: Mann-Whithey U-test, MDFS-1D, and MDFS-2D. For each filter, SGoF correction for multiple tests [51] was applied. This correction is optimised for the power of the test rather than for the reduction of false positive results. Its application assured a reasonable number of input variables for the machine learning algorithms. The number of selected biomarkers for various data sets and filtering methods is presented in Table 1. GE, RNA, METH contain over a thousand uncorrelated informative variables, hence the predictive models were built on 100 top-ranked ones. The number of selected features for CNA and RPPA data is much smaller, mainly due to stronger correlations between the variables. The sets of selected variables were stored for each fold of the cross validation. As shown in Table 1, the number and ranking of variables deemed relevant varied strongly between the folds of the cross validation procedure for both types of cancer.

**Table 1:** The number of all biomarkers for breast cancer (BRCA) and urothelial bladder carcinoma cancer (BLCA): the number of uncorrelated biomarkers in the entire dataset (column All), the number of uncorrelated informative identified by various FS methods (U-test, MDFS-1D, MDFS-2D)

Data	Dataset	All	Samples		FS methods		
					U-test	MDFS-1D	MDFS-2D
BRCA	GE	8673	entire		3674	3177	3905
			CV	total	6274	2644	3594
				mean	809	804	793
	CNA	22544	entire		87	131	173
			CV	total	342	908	1156
				mean	16	34	28
BLCA	RNA	19006	entire		2994	2710	3549
			CV	total	3419	6714	11826
				mean	525	626	592
	METH	15628	entire		1723	1619	1917
			CV	total	2898	6298	10351
				mean	474	469	482
	RPPA	219	entire		30	29	15
			CV	total	108	143	207
				mean	23	17	24

For each data set three rows correspond to the results obtained when filter was applied to the entire data set, the total number of uncorrelated informative biomarkers deemed relevant at least once in 150 repeats of cross-validation, and the average number of biomarkers for a single fold

For example, in RNA dataset for BLCA cancer, the total number of variables deemed relevant in 150 repeats of cross-validation was 11826, which is more than half of the total number of variables. Contrastingly, the average number for a single fold is only 592. This divergence arises due to the application of the SGoF procedure for the control of multiple tests. This procedure maximizes the power of the test, but allowing about ten per cent fraction of false positives. These false positives are different in different repeats of cross validation, leading to a large number of variables that were at least once identified as relevant. The feature sets selected with different methods are also quite divergent in examined data sets.

Nevertheless, models developed on different feature sets give comparable results. This is due to a high correlation between variables and the application of a greedy algorithm for selection of a representative of a cluster of similar variables. Small variations in ranking of the features may lead to a different choice of representatives. Nevertheless, the information about the decision variable remains similar for each choice. Therefore, the final predictive model is stable and does not depend on particular input features. This effect is well-known for omics data [52].

### Individual models

First, the molecular data sets and clinical data were studied independently. Generally, different feature filtering methods did not significantly alter the quality of the predictive models. Nevertheless, for some molecular data sets the application of the U-test as the filtering algorithm gave worse results in comparison with MDFS-1D or MDFS-2D. The MDFS-1D is more efficient computationally in comparison with MDFS-2D. Therefore, it was used in the subsequent analyses.

The results for the random forest models built with features selected by the Boruta algorithm for clinical data, and features selected by MDFS-1D method for molecular data are shown in three upper rows of Table 2 for BRCA data, and in four upper rows of Table 3 for BLCA data.

**Table 2:** The results of random forest models trained on BRCA data obtained with different sets of variables

Data set	ACC	MCC	AUC	*t* statistic	*p*-value
CD	0.677	0.362	0.739	–	–
GE	0.618	0.261	0.681	–	–
CNA	0.579	0.203	0.634	–	–
CD+GE	0.684	0.370	0.749	10.2 (30)	2.0 ⋅ 10^− 11^
CD+CNA	0.675	0.356	0.740	1.3 (17)	9.6 ⋅ 10^− 02^
CD+GE+CNA	0.685	0.368	0.753	10.4 (30)	2.0 ⋅ 10^− 11^
Super learner	**0.690**	**0.381**	**0.755**	**14.5** (30)	**4.2** ⋅ 10^− 15^

The upper panel displays results obtained with homogeneous data: clinical data (CD), gene expression (GE), copy and number aberrations (CNA). The middle panel displays results obtained for clinical data combined with the classification results of the molecular data (CD + GE, CD + CNA and CD + GE + CNA). The last row of the table displays the results of super learning procedure using NNLS method. The average values for ACC, AUC, and MCC are shown for all models. The last two columns show the results of the paired *t*-test between AUC values of integrated models compared with one obtained for clinical data only. The number in parentheses shows in how many times the integrated model was better than the baseline CD model in 30 repeats of cross validation, the best values are displayed in bold face

**Table 3:** The results of random forest models trained on BLCA data data obtained with different sets of variables

Data set	ACC	MCC	AUC	*t* statistic	*p*-value
CD	0.626	0.252	0.676	–	–
RNA	0.616	0.232	0.657	–	–
METH	0.576	0.154	0.623	–	–
RPPA	0.591	0.178	0.632	–	–
CD+RNA	0.642	0.279	0.690	4.2 **(25)**	1.2 ⋅ 10^− 4^
CD+METH	0.625	0.247	0.674	-0.81 (13)	7.9 ⋅ 10^− 1^
CD+RPPA	0.627	0.250	0.678	0.41 (19)	3.4 ⋅ 10^− 1^
CD+RNA+METH	0.635	0.255	0.683	2.2 (19)	1.6 ⋅ 10^− 2^
CD+RNA+RPPA	**0.644**	**0.282**	0.691	3.2 (24)	1.8 ⋅ 10^− 3^
CD+METH+RPPA	0.623	0.242	0.671	-1.1 (12)	8.6 ⋅ 10^− 1^
CD+all	0.638	0.265	0.685	2.2 (22)	1.7 ⋅ 10^− 2^
Super learner	0.625	0.268	**0.696**	**7.5** (23)	1.5 ⋅ 10^− 8^

The upper panel displays the results obtained with homogeneous data: clinical data (CD), RNA-seq (RNA), methylation (METH), reverse phase protein array (RPPA). The middle panel displays the results obtained for clinical data combined with the classification results of the molecular data (CD + RNA, CD + METH, CD + RPPA, CD + RNA + METH, CD + RNA + RPPA, CD + METH + RPPA, CD + RNA + RPPA + METH labelled as CD+all). The last row of table displays the results of super learning procedure using NNLS method. The average values for ACC, AUC, and MCC are shown for all models. The last two columns show the results of the paired *t*-test between AUC values of integrated models compared with one obtained for clinical data only. The number in parentheses shows in how many times the integrated model was better than the baseline CD model in 30 repeats of cross validation, the best values are displayed in bold face

While these results are far from perfect, they clearly show that all data sets contain significant information on the clinical endpoint for the patients. For both types of cancer, the best classification results were obtained for models which used clinical data. Models using molecular data exhibit significantly lower predictive power.

For breast cancer, models built using the CNA data set were the weakest, although still statistically significant. Such a result could have been expected in light of our understanding of the biological processes in cancer. The alteration of the number of copies of genes results in modified expression patterns in cells, that in turn can lead to the development of lethal forms of cancer. Nevertheless, each of these steps is mostly non-deterministic and depends on the individual history of the patient. Hence, the most information is contained on the clinical level, less on the gene expression level, and even less on the genetic alterations level.

For the bladder cancer, the highest prediction power (*AUC* = 0.657) among the molecular data sets was obtained from RNA data. The weakest model was constructed with RPPA data, probably due to a small number of informative variables (between 17 and 24).

### Combined models

#### Early integration strategy

Next, we examined, whether extending clinical data with molecular data can lead to an improved predictive power of machine learning models. The direct extension of the CD data set by adding the most relevant features from molecular data sets did not lead to better models. This occurs because the individual molecular features carry very little information in comparison with any of the clinical features. Consequently, they are very seldom used by a random forest classifier and have no influence on the final predictions of a model.

#### Late integration strategy

In the next part of the analysis, the advanced ensemble classification called super learning was used for prediction endpoints of cancer patients. The super learning model was built for both types of cancer using all the individual machine learning models based on clinical and molecular data with various feature filters. Three methods of combining various prediction results via super learner ensemble algorithm were tested, namely non-negative least squares (NNLS), random forest, and best-*k*. The non-negative least squares combining method proved to give the best results for both cancer types.

The values of evaluation metrics for super learning models are displayed in the last rows of Table 2 for the BRCA data and Table 3 for the BLCA data. For both cancer data sets, super learning predictive models outperform the individual machine learning models. The improvement is small but statistically significant.

For the BRCA data set, both the AUC and MCC have improved in all or all but one repeats of the procedure (p-values are 9.3 ⋅ 10^− 9^ and 4.2 ⋅ 10^− 15^, respectively).

For the BLCA data set, the results were weaker. The AUC has improved in 23 out of 30 repeats of the procedure. Nevertheless, the improvement of the AUC is statistically significant, with p-value from the paired t-test at 1.5 ⋅ 10^− 8^).

#### Hybrid integration strategy

In the hybrid model, the set of clinical descriptors was extended by the composite features, corresponding to the fraction of votes for the *deceased* class in random forest classifier built from molecular data sets. Adding all the combinations of synthetic molecular variables to the clinical ones was tested. The results of random forest combined models built on the extended data sets are displayed in rows 4-6 of Table 2 and the rows 5-11 of Table 3 for the BRCA data and the BLCA data, respectively.

For BRCA patients, only gene expression data contributes additional information on the clinical endpoint. However, the improvement of classification is visible only when using AUC as a quality metric. In particular, AUC improved in all 30 repeats of cross-validation, (p-value 9.3 ⋅ 10^− 9^). On the other hand, changes in MCC and ACC are minor and inconsistent. The CNA data does not contribute new information, all metrics either decrease (ACC and MCC) or increase minimally (AUC).

Similar results are obtained for BLCA patients: only knowledge of gene expression added some information to the clinical descriptors. This was reflected by changes in the AUC measure, which has improved 25 times in 30 repeats of cross-validation (p-value 1.6 ⋅ 10^− 4^). The predictive models did not improve when either methylation or RPPA data were added.

Both for BRCA and BLCA data sets, the best models obtained with the hybrid approach have comparable predictive power to the models obtained with the super learning. The best AUC was obtained with the super learning for both data sets, but the difference is tiny and not statistically significant. On the other hand, the best ACC and MCC were obtained with the super learner approach for BRCA, while for the BLCA the best results were obtained by the hybrid model with synthetic RNA and RPPA variables.

### Sensitivity analysis

The final step of this study was the sensitivity analysis. It was conducted on the combined CD + GE + CNA model for BRCA data, and CD + RNA + METH + RPPA model for BLCA data. In these models each molecular data set was represented as a single feature – the fraction of trees that predict that given patient belongs to the deceased class.

In the first type of sensitivity analysis, a single feature was removed from the description, and the decrease of the AUC of the model was used as a measure of importance. The second type of performed sensitivity analysis was the recursive feature elimination, where the least important features are iteratively removed from the predictive model.

**Fig. 4:**
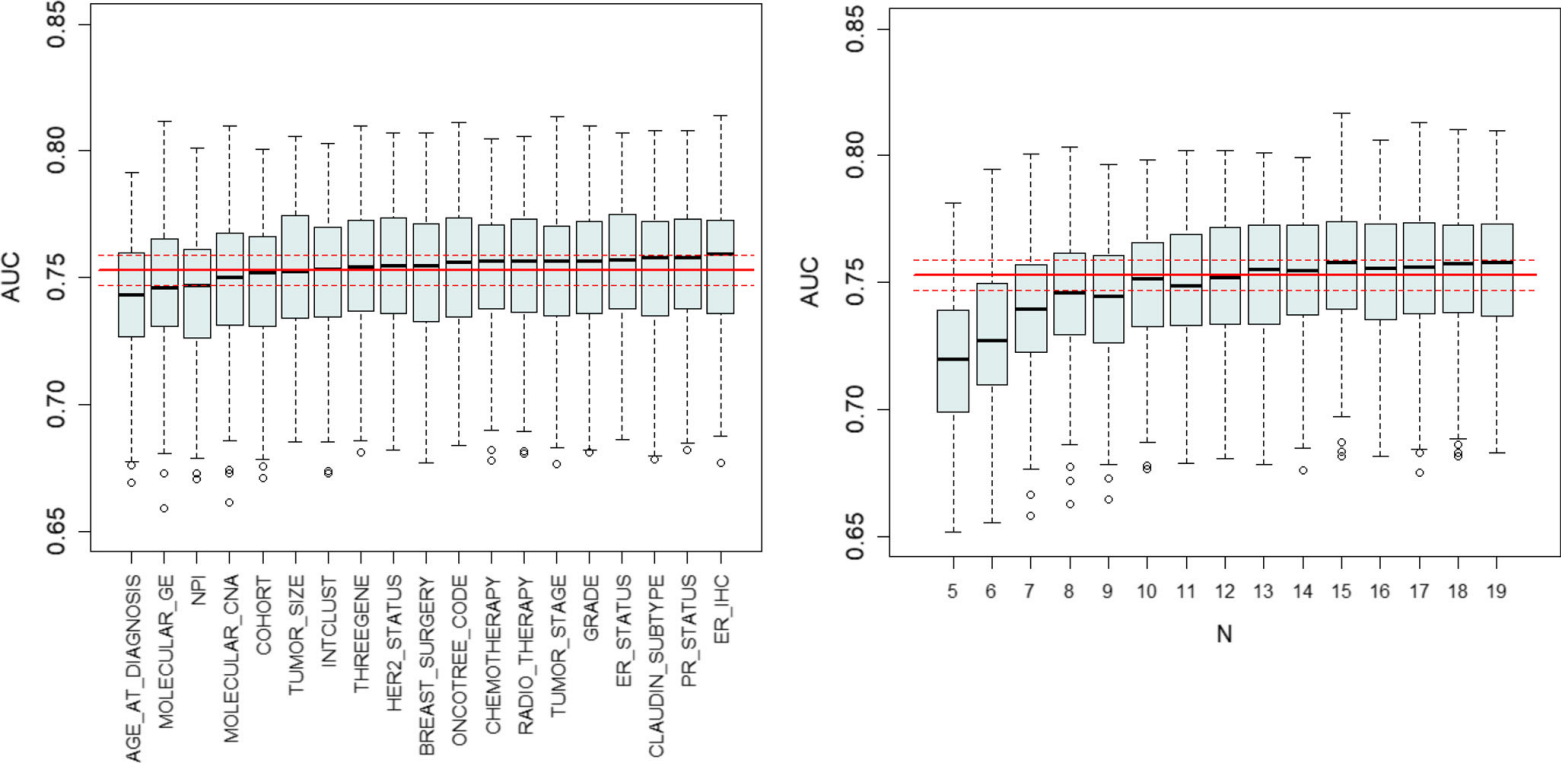
Results of sensitivity analysis for the BRCA data. Left panel: the change of AUC of the predictive model (CD + GE + CNA) after the removal of particular features. Right panel: The result of the recursive feature elimination. Red line indicates the level of AUC for the classifier built on top-17 clinical descriptors and two synthetic molecular descriptors. Dotted lines mark the standard deviation of ΔAUC

The first type of analysis conducted on the BRCA data has shown that molecular features representing both GE and CNA data, are relatively strong, see the third and fourth position of features in Fig. 4 (left panel). What is more, the inclusion of these features increased the robustness of the model, with respect to the removal of the features from the description. For the purely clinical model, the removal of any descriptor resulted in decreased accuracy. Contrastingly, after adding synthetic molecular features the significant decrease of AUC is present only for two clinical variables (*age at diagnosis*, and *NPI*) as well as for *molecular_GE*. This result suggests that molecular descriptors can replace clinical descriptors in a model, making it simpler and easier to interpret. This hypothesis is confirmed by the results of the RFE procedure, as shown on the right panel of Fig. 4. In this case, the quality of the model is stable until ten features are left in the description, namely *age at diagnosis, NPI, molecular_GE, molecular_CNA, cohort, intclust, tumor size, breast surgery, chemotherapy*,and *tumor stage*.

**Fig. 5:**
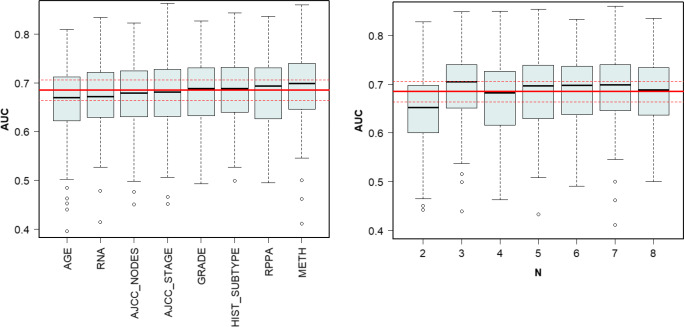
The results of sensitivity analysis for the BLCA data. Left panel: the change of AUC of the predictive model (CD + RNA + METH + RPPA) after the removal of particular features. Right panel: The result of the recursive feature elimination. Red line indicates the level of AUC for the classifier built on top-5 clinical descriptors and three synthetic molecular descriptors. Dotted lines mark the standard deviation of ΔAUC

Adding molecular descriptors had higher effect on the BLCA models. The extended model was resistant to removal of any single descriptor, see left panel of Fig. 5. What is more, the RFE procedure resulted in the best predictive model (*AUC* = 0.694) constructed using only three features: two clinical features (*age, ajcc_stage*) and one synthetic (*RNA*), see right panel of Fig. 5. An alternative model, using (*age, ajcc_nodes* and *RNA*) features (*AUC* = 0.696) can also be used, since *ajcc_nodes* and *ajcc_stage* are strongly dependent on one another, see Fig. 3.

## Conclusions

A new method, i.e. the hybrid data integration strategy, was introduced for the integration of heterogeneous types of data sets (clinical and omics data). The method uses results of machine learning predictions based on molecular data sets as new variables, which are then analyzed together with clinical ones. The approach is general and may be implemented using various machine learning algorithms. However, the use of random forest algorithm allowed to simplify the procedure thanks to its built-in OOB performance estimation.

The hybrid data integration protocol was applied to predict clinical endpoint for two types of cancer. In both cases adding a synthetic variable based on gene expression levels lead to a statistically significant increase in the predictive power of the combined model. On the other hand, the effects for synthetic variables based on other types of molecular data were not statistically significant. In particular, adding variables derived from methylation and RPPA data to the BLCA models seemed to decrease the quality of the models, although the effect is not statistically significant. Nonetheless, while the addition of variable derived from CNA data to BRCA models did not improve the predictive ability, it has contributed to the robustness of the model upon removal of variables from the description. The synthetic molecular variables performed as indicators of the progress of the disease on the molecular level. Their relative importance in combined models was high (second and fourth most relevant variable in the BRCA model and second most relevant variable for BLCA model).

The inclusion of the synthetic variables based on the RNA expression levels and copy number alterations can lead to an improved quality of prognostic tests. Thus, it should be considered for inclusion to medical practice.

The performance of the hybrid data integration approach was compared with two state-of-the-art methods of data integration: early integration and super learning. The new method proved to perform nearly as well as super learning and much better than the early integration, which was ineffective for this purpose. What is more, it gives a better understanding of results than super-learning. It also allows for reduction of model complexity.

## Data Availability

Data supporting the findings presented in this study is available in cBioPortal at https://www.cbioportal.org

## Code availability

The code is available on request from the corresponding author, A.P-K.
